# The Influence of Heredity and Contagion on the Propagation of Tuberculosis, and the Prevention of Injurious Effects from Consumption of the Flesh and Milk of Tuberculous Animals

**Published:** 1884-07

**Authors:** 


### REVIEWS.

The Influence of Heredity and Contagion on the Propagation of Tuberculosis, and the Prevention of Injurious Effects from Consumption of the Flesh and Milk of Tuberculous Animals. By Herr A. Lydtin, Carlsruhe, Adviser to the Baden Government; G. Fleming, LL.D., F.R.C.V.S., Principal Veterinary Surgeon to the British Army; and M. Van Hertsen, Veterinary Surgeon and Chief Inspector of the Brussels Abattoir. London: Baillière, Tindall & Cox; New York: Jenkins.

No more timely publication has recently appeared than this interesting volume. Since it deals with questions of vital importance to the physician and the veterinary surgeon, and emanating as it does from highly competent authorities, it cannot fail to be productive of practical results of a decidedly beneficial character. The hereditary nature of tubercular diseases has been long and sorrowfully known, but the facts leading to establish its contagious character have never before been brought in such array as to justify a positive conclusion. So long as any doubt touching this important question lurked in men's minds, so long might we expect that sanitary regulations looking to the prevention of such contagion would fail to receive adequate enforcement, and that only very imperfect measures would be taken to prevent the spread of this most lingering and fatal of diseases. But now that facts which admit of no cavil, conclusively prove that tuberculosis is in various ways communicable by contagion, we may hope that effective steps will be taken both by the public authorities and those who are largely interested in stock-raising, to stamp out the blighting tubercular diathesis. The difficulties of early recognizing the symptoms of tubercular disease, especially in cattle, have long stood in the way of establishing its contagious character, and the authors of the present work first wisely set about the task of pointing out the signs by which its insidious approaches may be detected. The confusion of views that has prevailed among veterinarians regarding the incipient phases of this disease, is well illustrated by the variety of names bestowed upon it by the writers of different nations. In order to put an end to this confusion, at once bewildering and misleading, the authors have adopted a definition of Tuberculosis lucid enough fully to describe the disease, and sufficiently elastic to meet the manifold shapes in which it occurs. They have called it "Tuberculosis panzöotica contagiosa." According to this definition, the nodosities, which are capable of being transmitted by inoculation to nearly all domesticated animals, and which, by their transmission, give rise to new nodosities, may in their turn be successfully inoculated. Wherever then these nodosities exist we have a case of true tuberculosis, and the kidney, heart, brain, and indeed any organ of the body, may be their nidus. The marked tendency which these nodosities exhibit to become deposited in the lungs, led many inquirers to confine their researches to those organs. This has been unfortunate, for it generally happens that the earliest manifestation of nodosities in the lungs is of short duration, and rapidly develops into pronounced acute tuberculosis; whereas in the other organs they remain latent for a much longer period of time. Thus when we meet them on the surface of the liver and spleen, or in the parenchyma of these organs, they fail to develop till general constitutional symptoms set in. The anatomical diagnosis of tuberculosis has therefore been a comparatively difficult matter to establish, and inquirers were often compelled to rely on an etiological diagnosis. Thanks, however, to the labors of Koch, it is now possible to recognize the nosogenic agent of tuberculosis in the very earliest stage of the malady, and to trace its existence even in the secreted fluids by the aid of the microscope, for the microbe of the disease having been brought to light, the existence of the disease necessarily results. This microbe follows the minutest channels, and enters the system as well by the digestive and genito-urinary passages as by the respiratory. It sometimes happens that the morbigenous agent is isolated or becomes encysted, and the evolution of the morbid process is either delayed or completely checked.

These and a host of other facts, conclusively established by the authors of the volume under consideration, go clearly to show the necessity of vigilantly noting the preliminary stages of the disease, since if there is any hope of arresting its progress, it must be at the very outset. What contributes chiefly to the propagation of tuberculosis, both by way of heredity and contagion, is the fact that it is not necessarily a fatal disease ; that so long as it does not attack an organ essential to the vegetative or animal existence, the animal may render all the services of one which is in a state of good health. Thus a cow may produce calves, furnish milk, etc. Such animals are an ever present source of danger to others, and so long as they are allowed to propagate and mix freely with others, there can be no hope of thoroughly eradicating this fell disease. The practical value, therefore, of an early recognition of the occult signs of tuberculosis, cannot be overestimated, and the authors have done an invaluable service for the profession in setting forth those signs and symptoms. Numerous instances are cited by them where the offspring of apparently healthy cattle exhibited almost from the moment of birth the undoubted evidences of tubercular disease, and this was the first circumstance that proved the mother was affected. Even then, in some cases at least, a post mortem examination alone revealed the existence of the trouble. Unfortunately, the too enthusiastic friends of sterpi-culture have unwittingly contributed to the maintenance of tuberculosis in its most deadly shape; for too fine breeding, which necessitates in and in breeding, produces a type exceedingly predisposed to tuberculosis. We must give up this too hot pursuit of perfect points in animals, and allow a coarser strain in our finest breeds if we would maintain their vigor and hope to eliminate the tubercular taint. This hereditary character of tuberculosis really constitutes a mere phase of its contagiousness, for transmission by propagation is nothing more than the infection of the ovum or fœtus through the medium of the parents. Of course it is contagion of a subtler sort, nor would we accept it as conclusive of the fact were not other arguments at hand. These arguments, however, are too numerous to admit of any question, and the authors have amassed an array of facts in proof of the contagious character of tuberculosis, which are of the utmost practical significance. Close cohabitation and the consequent respiration of the same air are the conditions of stable life; and in every instance where a pronounced case of tuberculosis shared such condition with animals entirely free from a tubercular diathesis, propagation by contagion resulted. Thus a veterinary surgeon in Waldkirch, duchy of Baden, witnessed tuberculosis develop in five bulls in the same stable. These bulls were not all related by blood, and four of them were considered absolutely sound when the five were brought together. The fifth, however, speedily showed signs of tubercular trouble, and before long the whole number succumbed to the disease. Tappeiner, in the experiments he made, the results of which awakened the keenest interest, found that the inhalation of matter expectorated by consumptive persons never failed in a very short period of time to generate pulmonary tuberculosis in dogs, and even to give rise to diffused miliary phthisis.

That careful and painstaking experimenter, Johne, has thus summed up the result of his patient investigations. 1st. The transmission of tuberculosis may be effected from animal to animal by the ingestion of tuberculous matters, but this mode of transmission is much more uncertain than by inoculation.

2d. The matters which most certainly transmit tuberculosis by gastro-intestinal ingestion, are those obtained from the lungs, the pleura, and lymphatic glands; the milk of tuberculous animals, with regard to its more or less certain action as contagious action, coming next to these. Infection takes place less readily with tuberculous matter obtained from mankind than with that from the lower animals.

3d. Infection takes place less readily by the ingestion of flesh than by means of the substances mentioned in No. 2.

4th. Calves, sheep, goats, and pigs present the greatest receptivity for the tubercular contagion.

In addition to these interesting conclusions, it may be stated that the experiments of Bollinger * have proved that the prolonged consumption of the milk of tuberculous cows produced miliary tuberculosis in the pig. These facts strongly favor the theory that tuberculosis is caused by the invasion of *bacilli*, and the experiments made by Koch corroborate this view. Having freed them from every heterogeneous element, and separated them from all other morbific agents, Koch introducd these bacilli into the sound animal system by way of inoculation, and obtained all the phenomena of tuberculosis. The utmost care was exercised in conducting these experiments, and all questionable material was diligently excluded. It was found that the bacilli obtained from human as well as from animal tubercle produced precisely the same results. This system of propagation by inoculation was indefinitely prolonged, nor did any signs of diminished vitality show itself in the latest bacilli. From his researches and experiments, Koch has drawn the following important conclusion, viz: "that the presence of bacilli in the tuberculous masses constitutes not only a concomitant fact in the tubercular process, but that it is the cause, and that we should see in the bacilli the cause of tuberculosis—a cause which had hitherto only been suspected, and which presents itself to us in the form of a vegetable parasite."

These facts prove the identity of animal and human tuberculosis; for Villemin and Krebs as well as Koch have demonstrated that tuberculous matter derived from man will produce pulmonary phthisis in animals, and this inoculated phthisis may be transmitted by inoculation to other animals. These conclusions are of the utmost value, since they unmistakably point to the only effectual means of contending with tuberculosis in its Protean shapes, and eventually stamping it out. Authorities are divided on the question how best to deal with suspicious animal food. Some, who wish to cheapen provisions for the poor, favor a lax system of supervision, holding that the thorough cooking of diseased meat fits it for use. It is true that bacilli are de-

* "Versamlung deutcher Natur forscher und Aerzte," Baden, 1879.

stroyed by long exposure to boiling water; but how many people, especially among the very class who are, by their circumstances, compelled to purchase inferior meat, are likely to observe this condition? A stringent enactment in regard to the consumption of questionable meat and milk is the only one which a wise legislature can adopt. Confiscation of diseased meat and the destruction of diseased animals are the only solution of the problem.

Space will not permit us to dwell at greater length on the topics discussed in this most recent and instructive monograph on comparative physiology and pathology, but its perusal must be eminently useful to those who are interested in questions affecting public health and in stock-raising.

C. M. O'L.

